# Simulated Gastric and Intestinal Fluid Electrolyte Solutions as an Environment for the Adsorption of Apple Polyphenols onto β-Glucan

**DOI:** 10.3390/molecules27196683

**Published:** 2022-10-08

**Authors:** Lidija Jakobek, Ivica Strelec, Daniela Kenjerić, Lidija Šoher, Ivana Tomac, Petra Matić

**Affiliations:** Faculty of Food Technology Osijek, Josip Juraj Strossmayer University of Osijek, Franje Kuhača 18, HR-31000 Osijek, Croatia

**Keywords:** dietary fiber, adsorption capacity, gastrointestinal tract, phenolic compounds

## Abstract

Interactions with dietary fibers in the gastrointestinal tract might affect the potential bioactivities of phenolic compounds. In this study, the interactions between apple phenolic compounds and β-glucan (a dietary fiber) were studied by studying the adsorption process in simulated gastric and intestinal fluid electrolyte solutions. Phenolic compounds were extracted from apples, adsorbed onto β-glucan (2 h, 37 °C, in gastric or intestinal fluid electrolyte solutions), and determined using high performance liquid chromatography. Phenolic compounds (flavan-3-ols, flavonols, phenolic acids, and dihydrochalcone) were stable in the gastric fluid (pH 3). In the intestinal fluid (pH 7), flavan-3-ols were not found and chlorogenic acid isomerized. Polyphenols from the apple peel (up to 182 and 897 mg g^−1^) and flesh (up to 28 and 7 mg g^−1^) were adsorbed onto β-glucan in the gastric and intestinal fluids, respectively. The adsorption was affected by the initial concentration of the polyphenols and β-glucan and by the environment (either gastric or intestinal fluid electrolyte solution). By increasing the initial polyphenol amount, the quantity of adsorbed polyphenols increased. Increasing the amount of β-glucan decreased the amount adsorbed. The results can be helpful in explaining the fate of phenolic compounds in the gastrointestinal tract.

## 1. Introduction

Phenolic compounds have shown a number of potential bioactivities. To explain these activities, knowledge of the fate of the compounds in the digestive system is of particular importance. It is well known that phenolic compounds are released during progress through different phases of digestion [1,2,3]. They can also be degraded in different parts of the digestive tract due to varying pH and environment [2,4]. Those that are absorbed go through phase I and phase II metabolism which creates different metabolites [5,6]. In addition, microorganisms present in the lower parts of the digestive tract create different catabolites of phenolic compounds [2,3,7]. All the mentioned processes and formed metabolites and catabolites might have an impact on the potential bioactivities of phenolic compounds and are currently the subject of intensive investigation.

Phenolic compounds can also interact with different food constituents, which might affect their passage through the digestive system, their absorption or degradation. For instance, phenolic compounds can interact with dietary fibers, which are not metabolized until they enter the colon [8]. Since they are not degraded, dietary fibers can “carry” intact phenolic compounds through the stomach and small intestine to the lower parts of the digestive tract (colon). In the colon, dietary fiber can be degraded, enabling phenolic compounds to be released and to show potential positive effects [9]. Although further research is necessary to confirm these effects, dietary fibers have previously been studied as colon-targeted delivery systems for polyphenols [10].

Interactions between phenolic compounds and dietary fiber can be studied in relation to the adsorption process [11]. In the adsorption process, a phenolic compound is the adsorbant and dietary fiber is the adsorbent. Molecules from the solution (phenolic compounds) adsorb onto the surface of the adsorbent (dietary fiber). The process can be described as a series of different steps: the transport of adsorbate in the solution to the adsorbent surface, the diffusion of adsorbate across the adsorbent liquid film, intra-particle diffusion, and adsorption and desorption from the surface of the adsorbent [12]. After reaching an equilibrium, the adsorption capacity *q*_e_, which is the amount of adsorbate adsorbed on the surface of the adsorbent, can be calculated. Some aspects of adsorption between phenolic compounds and various dietary fibers have been investigated previously [13,14]. β-glucan is a dietary fiber occurring in cereals, such as barley, and exhibits many positive bioactivities [15,16]. Phenolic compounds, such as epigallocatechin-3-gallat [13], tea polyphenols [17], phenolic acids [11], and quercetin derivatives [18] adsorb onto β-glucan. Due to the presence of different phenolic compounds in apples (e.g., flavan-3-ols, phenolic acids, dihydrochalcones, flavonols and anthocyanins) [19,20] and their potentially positive effect on human health [21,22], in previous investigations, we studied the adsorption of phenolic compounds from apples onto β-glucan in buffered solutions of pH 5.5 [23,24].

To the best of our knowledge, there have been no previous studies on the adsorption process between β-glucan and phenolic compounds from apples in simulated gastric and intestinal fluid electrolyte solutions. These fluids are commonly used in in vitro simulated gastric and intestinal digestion processes, processes which are important for the fate of phenolic compounds in the gastrointestinal tract. Gastric and intestinal fluid electrolyte solutions contain various electrolytes. The pH is usually adjusted to 3 (gastric fluid electrolyte solution) and 7 (intestinal fluid electrolyte solution). The adsorption and/or interaction between phenolic compounds and dietary fibers may be affected by the pH of the surrounding gastric and intestinal fluids. The chemical structures of phenolic compounds and dietary fibers at different ranges of pH may be different and could affect adsorption [25].

The purpose of this study was to investigate the adsorption of phenolic compounds from apples onto β-glucan in simulated gastric and intestinal fluid electrolyte solutions, to provide insight into the interactions between phenolic compounds and β-glucan. To this end, phenolic compounds were extracted from apple peel and flesh. The adsorption onto β-glucan was performed in a simulated gastric fluid electrolyte solution (2 h, 37 °C, pH 3) and in a simulated intestinal fluid electrolyte solution (2 h, 37 °C, pH 7). These time-periods and temperature were chosen since the gastric and intestinal phases of in vitro simulated digestion are usually studied for 2 h at 37 °C.

## 2. Results and Discussion

### 2.1. Identification of Phenolic Compounds from Apple Peel

**Figure 1:**
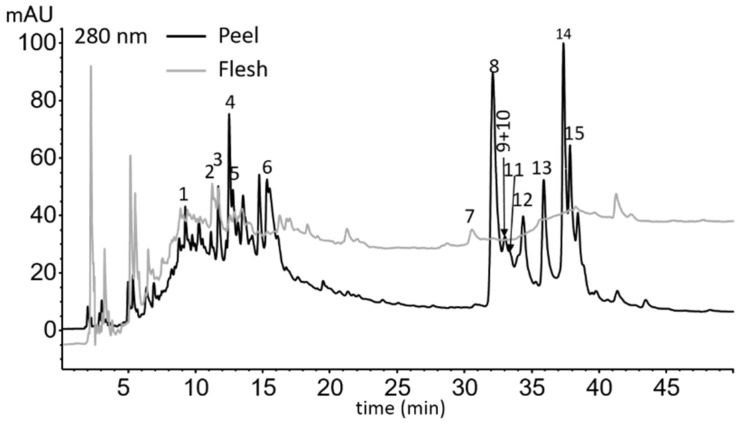
Chromatogram of apple peel and flesh extract before adsorption, scanned at 280 nm, with all detected phenolic compounds. Detected phenolic compounds. 1—procyanidin B1, 2—(+)-catechin, 3—procyanidin B2, 4—chlorogenic acid, 5—chlorogenic acid isomer 2*, 6—(−)-epicatechin, 7—phloretin-2-xyloglucoside*, 8—quercetin-3-galactoside, 9 + 10—quercetin-3-glucoside + quercetin-3-rutinoside*, 11—quercetin derivative 1*, 12—quercetin derivative 2*, 13—quercetin derivative 3*, 14—quercetin-3-xyloside, 15—quercetin-3-rhamnoside. *—tentative identification.

The maxima of the UV/Vis spectra of phenolic compounds detected in apple peel extract before and after adsorption in gastric fluid electrolytes are shown in Table S1. An example chromatogram of detected compounds from the peel is shown in Figure 1. Before adsorption, the phenolic compounds detected in the peel were flavan-3-ols (procyanidin B1, (+)-catechin, procyanidin B2, and (−)-epicatechin), phenolic acids (chlorogenic acid, and chlorogenic acid isomer 2), and flavonols (galactoside, glucoside, rutinoside, xyloside and rhamnoside of quercertin and three quercetin derivatives). The same compounds were detected after adsorption in the simulated gastric fluid.

After adsorption in the intestinal fluid electrolyte solution, some differences were observed (Table S1). Flavan-3-ols were not detected, chlorogenic acids were the same as before digestion (i.e., chlorogenic acid, and chlorogenic acid isomer 2), together with one new detected peak of phenolic acid (phenolic acid isomer 1). The flavonols were the same as those detected before the adsorption (i.e., galactoside, glucoside, rutinoside, xyloside and rhamnoside of quercetin and three quercetin derivatives). A new peak of phenolic acid can be seen in the chromatogram scanned at 320 nm, shown in Figure S1A, together with the already detected peaks of chlorogenic acids. Their UV/Vis spectra can be seen in Figure S1B.

The pH value of the simulated gastric and intestinal fluid electrolytes was different. Gastric fluid electrolyte solution had a pH value of 3 while in the intestinal fluid electrolyte solution the pH value was 7. The pH might have affected the chemical structures of the phenolic compounds and produced differences in the detected compounds after adsorption in the gastric or intestinal fluids. To identify which chemical forms phenolic compounds occur in at a certain pH, the pK_a_ value and the distribution diagram of species can be helpful. The pK_a_ value is the negative base-10 logarithm of the acid dissociation constant. At pH lower than pK_a_, the molecule exists mostly in a non-dissociated form, which is the form usually determined with HPLC, while at pH higher than pK_a_, the molecule exists in dissociated forms. At a pH value that is equal to pK_a_, 50% of the compound is in a dissociated form and 50% in a non-dissociated form. The distribution diagram of a molecule shows the relationship between the relative fractions of different species and the pH. Herrero-Martinez et al. (2005) [26] determined the pK_a_ values of some flavan-3-ols, such as catechin and epicatechin (catechin pK_a_ 8.77, 9.97, 11.99; epicatechin pK_a_ 9.00, 10.20, 12.20), and flavonols, such as quercetin (quercetin pK_a_ 7.19, 9.36, 11.56). According to the observed pK_a_ values for flavan-3-ols and flavonols, it appears that flavan-3-ols and flavonols exist in non-dissociated forms at pH value 3. The pK_a_ values of chlorogenic acids were also determined (chlorogenic acid pK_a_ 3.44; neochlorogenic acid pK_a_ 3.46) [27]. Chlorogenic acid had a lower pK_a_ value (3.4) [27], but this still indicated that, at pH 3, this compound can be present in a non-dissociated form in high amounts (~70%). In addition, a study by Narita and Inouye (2013) [28] indicated that chlorogenic acid was stable at lower pH and did not isomerize. The flavan-3-ols, flavonols and phenolic acids identified after adsorption in the gastric fluid were in a non-dissociated form, the same form as the compounds before adsorption. This was the reason for identifying the same compounds before and after adsorption in the gastric fluid.

However, at pH 7, which is the pH of the simulated intestinal fluid electrolyte solution, the pH could affect some compounds. According to their pK_a_ values [26], flavan-3-ols could still be present in their non-dissociated forms. The distribution diagram published in the literature of catechin [26], one of the representatives of flavan-3-ols, confirms that, at pH 7, catechin can be present in the original non-dissociated chemical form. However, some studies have reported the degradation of flavan-3-ols in the intestinal fluid [4,29]. In these studies [4,29], apples were subjected to a simulated digestion process—the concentrations of flavan-3-ols after the intestinal digestion phase were much lower than in the preceding gastric digestion phase, indicating degradation. Since the pK_a_ values indicate the presence of non-dissociated forms of flavan-3-ols at pH 7, and some studies point to their degradation in the intestine, the fate of flavan-3-ols is not clear. It is possible that, in this study, the flavan-3-ols degraded or were completely adsorbed, which resulted in their absence after adsorption in the intestinal fluid. In addition, quercetin, as a representative of flavonols, has a pK_a_ value of 7.19 [26]; however, the distribution diagram published in the literature [26] indicates that, at pH 7, the original non-dissociated molecular form is present in high amounts (~60%) [26]. Moreover, earlier studies have suggested that flavonols are stable during intestinal digestion [4,29]. The fact that flavonols, such as aglycone quercetin, exist in non-dissociated form, and that they are stable, suggests that flavonols from apples which are quercetin glycosides can exist in a non-dissociated form in the intestinal fluid at pH 7. This explains why the same flavonols were detected before and after adsorption in the intestinal fluid. However, phenolic acids can change. According to its pK_a_ value, chlorogenic acid can dissociate at pH 7. In addition, chlorogenic acid or 5-caffeoylquinic acid can isomerize into 3-caffeoylquinic acid and 4-caffeoylquinic acid, depending on the pH of the environment and the elapsed time [28]. More precisely, at pH 5 to 5.5, 5-caffeoylquinic acid isomerizes to 4-caffeoylquinic acid, and between pH 6 and 9 it isomerizes to both 3-caffeoylquinic acid and 4 caffeoylquinic acid [28]. Accordingly, the possible isomerization of chlorogenic acid in the intestinal fluid to 3- and 4-caffeoylquinic acid is suggested in our study. This may explain why one additional peak (phenolic acid isomer 1) was detected after adsorption in the intestinal fluid. The isomerization of chlorogenic acid in the intestinal fluid was suggested in an earlier study where the bioaccessibility of phenolic compounds from apples was investigated with in vitro simulated digestion [4]. In short, after adsorption in the intestinal fluid electrolyte solution, degradation or complete adsorption might explain the absence of flavan-3-ols, and isomerization of chlorogenic acid might be the cause of the additional peak observed for phenolic acid. Flavonols might be in non-dissociated forms, the same forms as before adsorption, and were all present after adsorption. However, these results need to be confirmed in additional studies.

### 2.2. Identification of Phenolic Compounds from Apple Flesh

An extract of the apple flesh before adsorption (Figure 1, Table S1) contained flavan-3-ols (procyanidin B1, (+)-catechin, and procyanidin B2), phenolic acids (chlorogenic acid, and chlorogenic acid isomer 2), and dihydrochalcones (phloretin-2-xyloglucoside). The same compounds were detected after adsorption in the gastric fluid electrolyte solution (Table S1), except for chlorogenic acid, which was not detected, possibly due to its complete adsorption. The presence of non-dissociated forms of flavan-3-ols, dihydrochalcone, and chlorogenic acids at the pH of the gastric fluid (pH 3) is suggested, as previously mentioned.

Some differences were observed in the compounds detected after adsorption in the intestinal fluid electrolyte solution (Table S1). Flavan-3-ols were not detected due to possible degradation, as already mentioned. Two phenolic acids identified were the same as before adsorption (chlorogenic acid, and chlorogenic acid isomer 2), but one new peak was detected (phenolic acid isomer 1). Dihydrochalcone was the same as before adsorption. The isomerization of chlorogenic acid in the intestinal fluid is possible.

### 2.3. Adsorption of Phenolic Compounds from Apples

**Figure 2:**
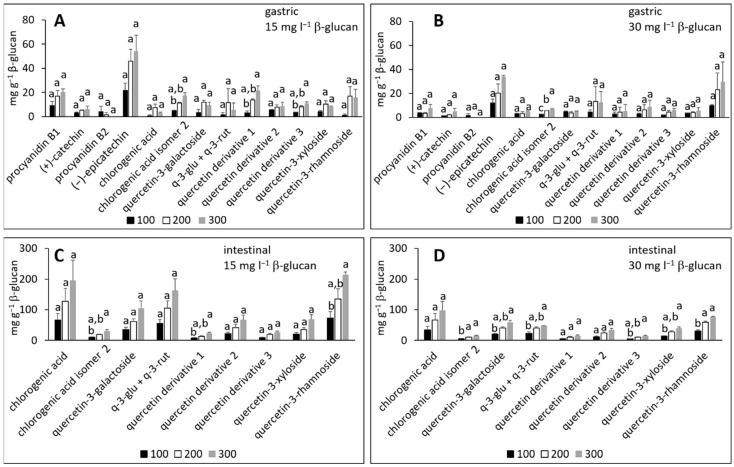
The amounts of individual phenolic compounds from apple peel adsorbed onto β-glucan (mg g^−1^ of β-glucan) in gastric fluid electrolyte solution with added (**A**) 15 mg L^−1^ β-glucan, (**B**) 30 mg L^−1^ β-glucan and in the intestinal fluid electrolyte solution with added (**C**) 15 mg L^−1^ β-glucan and (**D**) 30 mg L^−1^ β-glucan. Volumes of phenolic compound extracts were 100, 200 and 300 μL. The significant differences between the amount adsorbed with different initial volumes of phenolic compound extract according to post-hoc Tukey test (*p* < 0.05) are marked by different letters above bars.

**Figure 3:**
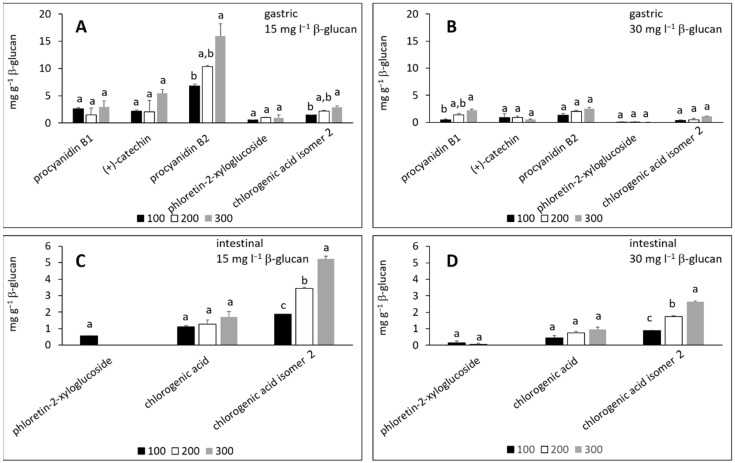
The amounts of individual phenolic compounds from apple flesh adsorbed onto β-glucan (mg g^−1^ of β-glucan) in gastric fluid electrolyte solution with added (**A**) 15 mg L^−1^ β-glucan, (**B**) 30 mg L^−1^ β-glucan, and in the intestinal fluid electrolyte solution with added (**C**) 15 mg L^−1^ β-glucan and (**D**) 30 mg L^−1^ β-glucan. Volumes of phenolic compound extracts were 100, 200 and 300 μL. The significant differences between the amount adsorbed with different initial volumes of phenolic compound extract according to post-hoc Tukey test (*p* < 0.05) are marked by different letters above bars.

In the simulated gastric fluid electrolyte solution, individual phenolic compounds from the peel (Figure 2A,B) adsorbed onto β-glucan whether β-glucan was added in 15 or 30 mg L^−1^, or the initial volume of the added phenolic extract was 100, 200 or 300 μL. The same outcome was observed for phenolic compounds from the flesh (Figure 3A,B), with individual compounds adsorbed in the presence of 15 or 30 mg L^−1^ of β-glucan. The adsorption capacities, expressed in mg per g β-glucan, were up to 54 (Figure 2A) and 34 mg g^−1^ β-glucan (Figure 2B) for compounds from apple peel, and up to 16 (Figure 3A) or 2.5 mg g^−1^ (Figure 3B) for compounds from apple flesh. Adsorption capacities depend on the pH value, the ionic strength, and the temperature [13,17,30], which makes it difficult to compare results obtained with those published in the literature. However, the adsorption capacities observed in this study were in accordance with our previous work [23], and with other studies [13,17]. For example, the adsorption capacities of β-glucan for different phenolic compounds from tea were 0.4 to 40 mg g^−1^ β-glucan [13], for different tea polyphenols from a standard mixture were from 156 to 405 mg g^−1^ β-glucan [13], and for tea polyphenols at different pH values were up to 116 mg g^−1^ β-glucan [17].

By increasing the initial volume of the phenolic compound extract from 100 to 300 μL, the amount of adsorbed phenolic compounds increased for most of the compounds (Figure 2A,B and Figure 3A,B). However, the increase was statistically significant only for some compounds. This agrees with an earlier study where the adsorption capacity of (−)-epigallocatechin-3-gallate adsorbing onto β-glucan increased with increase in the initial concentration of a compound [13].

As mentioned earlier, phenolic compounds could be present in different forms at different pH values. At pH 3, the pH of the gastric fluid electrolyte solution, flavan-3-ols, flavonols, dihydrochalcones and chlorogenic acids were probably present in non-dissociated forms. The bonds that could be created between non-dissociated forms of phenolic compounds and β-glucan are H bonds. Phenolic compounds and β-glucan possess many OH groups that could be responsible for H bonding. The creation of non-covalent bonds, such as H bonds and van der Waals forces, between phenolic compounds and β-glucan has been suggested previously [13,17,30,31] and agrees with our suggestion. Although hydrophobic interactions are also possible, due to hydrophobic aromatic rings in phenolic compound molecules, hydrophobic interactions might not be so common [17].

After adsorption in the simulated intestinal fluid electrolyte solution (Figure 2C,D and Figure 3C,D), flavan-3-ols from the peel or flesh could not be detected. Other compounds adsorbed onto β-glucan whether β-glucan was added in amounts of 15 or 30 mg L^−1^. The adsorption capacities were up to 214 (Figure 2C) and 98 mg g^−1^ β-glucan (Figure 2D) for compounds from apple peel, or up to 5.3 (Figure 3C) or 2.6 mg g^−1^ (Figure 3D) for compounds from apple flesh. Again, the adsorption capacities increased with increased initial volume of the extract from 100 to 300 μL. The increase was statistically significant only in some cases, consistent with other studies [13,17,23].

At the pH level of the intestinal fluid (pH 7), flavan-3-ols might degrade, which could be the reason for their absence. Flavonols and dihydrochalcones might be present in non-dissociated forms, while chlorogenic acids can isomerize and dissociate [28]. However, H bonds may account for the adsorption of flavonols, dihydrochalcones, and phenolic acids onto β-glucan due to OH groups present on the molecules.

### 2.4. Total Adsorption Capacities

**Figure 4:**
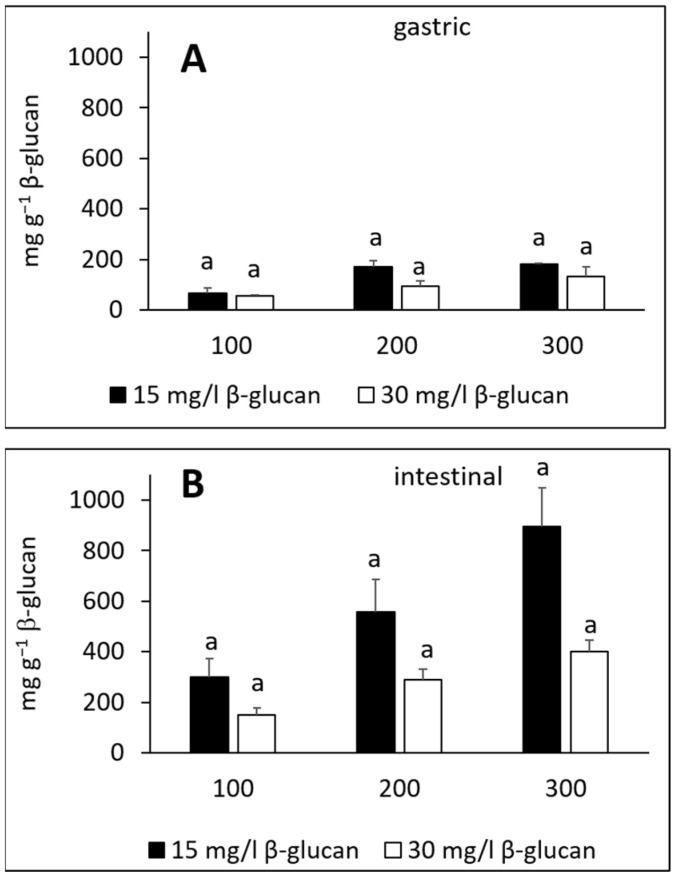
Total amount of adsorbed phenolic compounds from apple peel onto β-glucan in simulated (**A**) gastric and (**B**) intestinal fluid electrolyte solutions. The amount of β-glucan was 15 and 30 mg L^−1^. Phenolic compound extract volumes were 100, 200 and 300 μL. The significant differences between the total amount of adsorbed phenolic compounds with different initial amount of β-glucan were analyzed with post-hoc Tukey test (*p* < 0.05), the same letter above bars shows no significant difference.

Addition of the amounts of individual phenolic compounds adsorbed onto β-glucan provides the total adsorbed amount. Figure 4 shows the total amount of adsorbed phenolic compounds from apple peel. Phenolic compounds adsorbed in the gastric fluid electrolyte solution (Figure 4A) in total amounts of 67–182 or 56–133 mg g^−1^ for β-glucan present in 15 or 30 mg L^−1^, respectively. In the intestinal fluid electrolyte solution (Figure 4B), the total adsorbed amounts were 300–897 or 151 to 401 mg g^−1^ when the initial β-glucan concentration was 15 or 30 mg L^−1^, respectively. The total amounts adsorbed were different for different initial amounts of β-glucan. They decreased as the initial amount of β-glucan increased, though the differences were not statistically significant. In addition, the total adsorbed amounts were higher in the intestinal fluid than in the gastric fluid.

**Figure 5:**
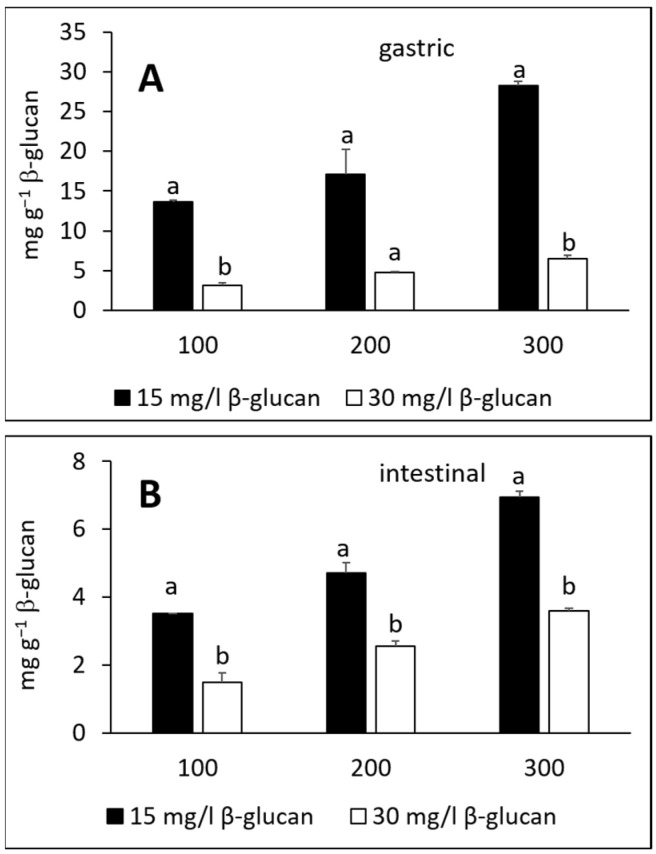
Total amount of adsorbed phenolic compounds from apple flesh onto β-glucan in simulated (**A**) gastric and (**B**) intestinal fluid electrolyte solutions. The amount of β-glucan was 15 and 30 mg L^−1^. Phenolic compound extract volumes were 100, 200 and 300 μL. The significant differences between the total amount of adsorbed phenolic compounds with different initial amount of β-glucan according to post-hoc Tukey test (*p* < 0.05) are marked by different letters above bars.

Figure 5 shows the total amounts of adsorbed phenolic compounds from apple flesh. In the gastric fluid electrolyte solution, the total adsorbed amounts were 14–28 or 3–6 mg g^−1^ for 15 or 30 mg L^−1^ initial β-glucan concentration, respectively (Figure 5A). In the intestinal fluid electrolyte solution, polyphenols adsorbed from 3–7 or from 2–4 mg g^−1^ for 15 or 30 mg L^−1^ initial β-glucan concentration, respectively (Figure 5B). Again, it was observed that the total adsorbed amount decreased as the initial amount of β-glucan increased, and here the differences were statistically significant. The adsorption capacities were lower in the intestinal fluid than in the gastric fluid.

The amount of β-glucan can affect adsorption, as shown in Figure 4 and Figure 5. A higher initial concentration of β-glucan results in lower amounts of phenolic compounds adsorbed per g of β-glucan. This might be due to a smaller contact area between the phenolic compounds and β-glucan when the concentration of β-glucan increases.

### 2.5. Principal Component Analysis

**Figure 6:**
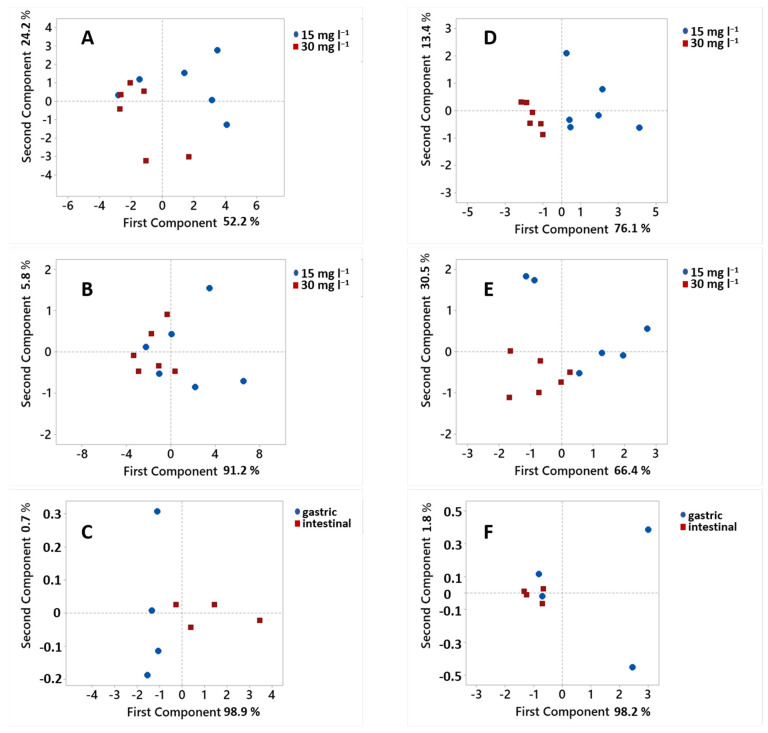
Principal component analysis of adsorption capacities of all individual phenolic compounds from apple peel (**A**) in the gastric solution, (**B**) in the intestinal fluid electrolyte solution. Principal component analysis of (**C**) total adsorption capacities of phenolic compounds from apple peel in both gastric and intestinal fluid electrolyte solutions. Principal component analysis of adsorption capacities of all phenolic compounds from apple flesh (**D**) in the gastric solution, (**E**) in the intestinal fluid electrolyte solution. Principal component analysis of (**F**) total adsorption capacities of phenolic compounds from apple flesh in both gastric and intestinal fluid electrolyte solutions.

Since differences were observed in the amount of adsorbed phenolic compounds due to different initial amounts of β-glucan or to adsorption in different simulated fluids, we performed additional analyses to confirm the results. The amounts of adsorbed compounds (*q*_e_) were analyzed using principal component analysis (PCA) to determine possible clustering of these data according to the different amounts of β-glucan or according to the use of different simulated fluids (Figure 6).

The PCA analysis of the amount of all adsorbed individual phenolic compounds from peel in the gastric (Figure 6A) and intestinal fluid electrolyte solutions (Figure 6B), and from flesh in the gastric (Figure 6D) and intestinal fluid electrolyte solutions (Figure 6E), showed clustering according to different amounts of β-glucan (15 or 30 mg L^−1^ β-glucan). This suggests that the adsorption capacities differed according to the presence of different amounts of β-glucan in the reaction solution. In the above analyses, the first two components accounted for 76.4, 97, 89.5 and 96.9% of the variation in the amounts adsorbed (Figure 6A,B,D,E, respectively). The clustering of data according to the different amount of β-glucan confirmed the results shown in Figure 4 and Figure 5.

The total amounts of adsorbed phenolic compounds from peel (Figure 6C) and from flesh (Figure 6F) in gastric and intestinal fluid electrolyte solutions were analyzed with PCA. Clustering according to the adsorption taking place in the gastric or intestinal fluid can be seen (Figure 6C). This suggests that adsorption might be different due to the use of different simulated fluids. However, there was no clustering for the phenolics from the flesh (Figure 6F). The first two components accounted for 99.6 and 100% of the differences.

### 2.6. Regression Analysis

Regression analysis was conducted to obtain additional insight into the observed differences. The amounts of all individual phenolic compounds from peel and flesh adsorbed onto β-glucan (mg g^−1^), as a function of different volumes of extracts (μL), were analyzed with simple regression analysis. For the relationship between the amounts adsorbed onto 15 mg L^−1^ of β-glucan vs. volume, and the amounts adsorbed onto 30 mg L^−1^ of β-glucan vs. volume, the regression lines were different, showing different slopes (coefficients). The slopes were statistically significantly different (Table S2). Similarly, for the relationship between the amounts adsorbed in the gastric fluid vs. volume, and the amounts adsorbed in the intestinal fluid vs. volume, the regression lines were again different. They showed significantly different slopes (coefficients) (Table S3). These analyzes confirmed that adsorption onto different amounts of β-glucan (15 or 30 mg L^−1^) and adsorption in different simulated fluids were significantly different.

A complete model was created using multiple variable regression. The variables were again taken to be the volume of the extract but were now multiplied by various indicators. These indicators were for the simulated fluid (gastric or intestinal electrolyte solution), the amount of β-glucan (15 or 30 mg L^−1^), the part of the apple (peel or flesh), and the type of phenolic compound, and certain interactions between these variables. The complete regression table containing the strongly significant coefficients is shown in Table S4. It has a standard deviation of residuals of 11.9 mg g^−1^.

The adsorption of phenolic compounds from apple onto β-glucan, or interactions between them, can be affected by the amount of β-glucan and by the conditions of the environment where adsorption takes place (e.g., gastric or intestinal fluid electrolyte solutions). A higher amount of β-glucan can lead to a lower amount of adsorbed phenolic compounds per mass of β-glucan. This observation might be important for the development of dietary fibers as carriers of phenolic compounds through the digestive tract to the colon. In addition, the environment, such as gastric and intestinal fluids, has an impact on the adsorption of phenolic compounds from apples.

## 3. Materials and Methods

### 3.1. Chemicals

Chemicals were purchased from several companies: Gram mol (Zagreb, Croatia): potassium dihydrogen phosphate, potassium chloride, sodium hydrogen carbonate, and magnesium chloride; Kemika (Zagreb, Croatia): ammonium carbonate; Carlo Erba Reagents (Val de Reuil, France): sodium chloride; Fluka (Buchs, Switzerland): orto-phosphoric acid 85% HPLC-grade; and J.T. Baker (Gliwice, Poland): methanol HPLC grade. Polyphenol standards ((+)-catechin hydrate, (−)-epicatechin, chlorogenic and *p*-coumaric acid, quercetin dihydrate, quercetin-3-rutinoside hydrate, quercetin-3-glucoside), and barley β-D-glucan, were purchased from Sigma-Aldrich (St. Louis, MO, USA). Procyanidin B1 and B2, phloretin and phloretin-2′-*O*-glucoside, quercetin-3-galactoside, quercetin-3-rhamnoside, and cyanidin-3-galactoside chloride were obtained from Extrasynthese (Genay, France).

### 3.2. The Preparation of Simulated Gastric and Intestinal Fluid Electrolyte Solutions

Electrolytes were prepared according to [32] in concentrations of 0.5 mol L^−1^ (KCl, KH_2_PO_4_, (NH_4_)_2_CO_3_), 1 mol L^−1^ (NaHCO_3_), 0.15 mol L^−1^ (MgCl_2_), and 2 mol L^−1^ (NaCl). Simulated gastric fluid electrolyte solution was prepared in a volumetric flask of 100 mL by pipetting electrolytes in a flask and adding distilled water to a final volume, with final electrolyte concentrations 8.625 mmol L^−1^ KCl, 1.125 mmol L^−1^ KH_2_PO_4_, 31.25 mmol L^−1^ NaHCO_3_, 0.15 mmol L^−1^ MgCl_2_, 0.625 mmol L^−1^ (NH_4_)_2_CO_3_, 59 mmol L^−1^ NaCl. The pH was adjusted to 3 with 1M HCl. Simulated intestinal fluid electrolyte solution was prepared in a volumetric flask of 100 mL with the final concentrations of electrolytes 8.5 mmol L^−1^ KCl, 1 mmol L^−1^ KH_2_PO_4_, 106.25 mmol L^−1^ NaHCO_3_, 0.4125 mmol L^−1^ MgCl_2_, and 48 mmol L^−1^ NaCl. The pH was adjusted to 7 with 1 M HCl.

### 3.3. Samples

Apples were purchased at the local market. One kilogram was peeled, flesh residue from the peel was scraped with a knife, and the peel was homogenized using a coffee grinder. The flesh was cut into quarters and the core was removed. The flesh was homogenized using a stick blender. Citric acid was added to the homogenized flesh to prevent enzymatic browning. The homogenized samples of flesh and peel were stored in the freezer at −18 °C for one night and used to extract phenolic compounds the next day.

### 3.4. Extraction of Phenolic Compounds

The peel was weighed (0.2 g) in six plastic tubes. An amount of 1 mL of distilled water was added to each tube, the tubes were put in an ultrasonic bath (Bandelin Sonorex RK 100, Berlin, Germany) for 15 min, and then centrifuged (Eppendorf, Hamburg, Germany) at 10,000 rpm for 10 min. Extracts were separated from residues and combined into one extract of a volume of 6 mL. Residues were extracted again with the same procedure using 0.5 mL of distilled water. The obtained extracts were combined and added to the earlier prepared 6 mL to obtain a final extract of total 9 mL volume. The procedure was repeated with the apple peel to obtain a second parallel extract. Phenolic compounds from flesh were extracted with the same procedure, and in two parallel procedures. One ml of each extract was filtered through 0.2 μm syringe filters (PTFE) and analyzed using HPLC immediately after preparation.

### 3.5. Adsorption

The adsorption of phenolic compounds onto β-glucan in the simulated gastric fluid electrolyte solution and in the simulated intestinal fluid electrolyte solution was conducted on the same day the extracts were prepared. The total volume of reaction solution in which the adsorption took place was 1.5 mL. The concentrations of β-glucan in the reaction solutions were 15 or 30 mg L^−1^, and the phenolic compound extracts were added in three different volumes, 100, 200, and 300 μL. For the adsorption in the simulated gastric fluid electrolyte solution, reaction solutions contained either 15 or 30 mg L^−1^ of β-glucan, different volumes of peel or flesh extract (100, 200, and 300 μL), and the remainder, to a total of 1.5 mL, was simulated gastric fluid electrolyte solution. In plastic tubes, the solutions were incubated for 2 h in the incubator (IN 30 Memmert, Schwabach, Germany) at 37 °C, and centrifuged for 10 min at 10,000 rpm. Then 1 mL of each reaction solution was filtered through a 0.2 μm PTFE syringe filter and analyzed immediately on an HPLC system. The determined amount was the amount of unadsorbed phenolic compounds (*c*_e_). The same experiment was conducted in the simulated intestinal fluid electrolyte solution.

The adsorption capacity of each individual phenolic compound (*q*_e_) (mg of phenolic compounds adsorbed onto g β-glucan) was calculated by the following equation:

(1) $$q_{e}=\frac{\left(c_{0}-c_{e}\right)V_{m}}{\gamma_{a}V_{a}}$$

*c*_0_ is the initial concentration of phenolic compounds after adding 100, 200 and 300 μL of extract in the reaction solution (mg L^−1^), *c*_e_ is the polyphenol concentration in the reaction solution after adsorption or unadsorbed polyphenols (mg L^−1^), *V*_m_ is the total volume of the reaction solution (l), *γ*_a_ is the β-glucan concentration (g L^−1^) and *V*_a_ is the volume of added β-glucan in the reaction solution (l)). The total adsorption capacity was obtained by adding the adsorption capacities of all individual compounds present in the extract.

As explained later in the text, a portion of the chlorogenic acid isomerized into phenolic acid isomer 1 after adsorption in the intestinal fluid. To correctly calculate the amount of adsorbed chlorogenic acid in the intestinal fluid (*q*_e_), the unadsorbed amount needs to be calculated correctly, according to equation 1. So, we calculated the amount of unadsorbed chlorogenic acid (*c*_e_) by adding the concentrations of chlorogenic acid found unadsorbed after adsorption and its created isomer–phenolic acid isomer 1, which was also found unadsorbed in the intestinal fluid. This *c*_e_ was used to calculate the amount of adsorbed chlorogenic acid (*q*_e_) in the intestinal fluid.

### 3.6. HPLC Analyses

Extracts from the peel and flesh of apples and samples obtained after adsorption were analyzed using the HPLC system 1260 Infinity II (Agilent technology, Santa Clara, CA, USA), consisting of a quaternary pump, a PDA detector, and a vial sampler. The column was a Poroshell 120 EC C-18 column (4.6 × 100 mm, 2.7 μm) protected with a Poroshell 120 EC-C18 4.6 mm guard-column. Individual phenolic compounds were separated with the flow of mobile phases 0.5 mL min^−1^ (A = 0.1% H_3_PO_4_ in water, B = 100% methanol) using a gradient: 0 min 5% B, 5 min 25% B, 14 min 34% B, 25 min 37% B, 30 min 40% B, 34 min 49% B, 35 min 50% B, 58 min 51% B, 60 min 55% B, 62 min 80% B, 65 min 80% B, 67 min 5% B, 72 min 5% B. Samples were injected in 10 μL volume. For the identification, we used spectra (200 to 600 nm) and retention times of authentic standards and compared them to those of the peaks in the samples. The amounts were obtained using calibration curves of authentic standards. Some phenolic compounds were tentatively identified as chlorogenic acid isomer 1 and 2, phloretin-2-xyloglucoside and quercetin derivatives, and were quantified using calibration curves of chlorogenic acid, phloretin, and quercetin, respectively.

### 3.7. Statistical Analyses

All extracts from the peel and flesh of apples were prepared in two parallel procedures, and each were measured two times on HPLC (*n* = 4). The adsorption experiment was conducted once with each parallel procedure (*n* = 2). The results were reported as means ± standard deviation of means. The differences between the adsorption capacities were analyzed with a post hoc Tukey test, with principal component analysis, and by regression analysis using Minitab software (Minitab LLC, State College, PA, USA).

## 4. Conclusions

This study showed that phenolic compounds from apples adsorb onto β-glucan in the environment created by simulated gastric and intestinal fluid electrolyte solutions. This was shown for the individual phenolic compounds from the peel, which adsorbed up to 54 or 214 mg g^−1^, and individual polyphenols from the flesh, which adsorbed up to 16 or 5.3 mg g^−1^ in the gastric or intestinal fluids, respectively. The total adsorbed amounts of the peel polyphenols were up to 182 and 897 mg g^−1^, and from the flesh up to 28 and 7 mg g^−1^ in the gastric and intestinal fluids, respectively. A higher initial quantity of phenolic compounds led to a higher amount of adsorption onto β-glucan. The regression analysis showed that adsorption with different initial amounts of β-glucan (15 and 30 mg L^−1^) was statistically significantly different. This indicated that adsorption was dependent on the initial β-glucan amount. A lower amount of β-glucan adsorbed more polyphenols per unit mass. The regression analysis showed that adsorption in different environments (gastric and intestinal fluids) was statistically significantly different. The adsorption was dependent on the environment where adsorption took place, i.e., gastric or intestinal fluid electrolyte solutions. Future work will be directed towards the identification of isomers and the degradation products of phenolic compounds for different pH values.

## Data Availability

Not applicable.

## Supplementary Materials

The following supporting information can be downloaded at: https://www.mdpi.com/article/10.3390/molecules27196683/s1, Table S1: Phenolic compounds from peel and flesh of apples detected before the adsorption and after the adsorption in the gastric and intestinal fluid electrolyte solutions, with maximums of UV/Vis spectra. Figure S1: (A) the chromatogram of apple peel after the adsorption in the simulated gastric and intestinal fluid electrolyte solutions scanned at 320 nm with detected phenolic acids. (B) UV/Vis spectra of phenolic acids. Detected phenolic acids. phenolic acid isomer 1*, 4—chlorogenic acid, 5—chlorogenic acid isomer 2*. *—tentative identification. Table S2: Regression analysis of the data of individual phenolic compounds adsorbed per mass of β-glucan (mg g^−1^). Comparison of gastric and intestinal fluid electrolyte solutions. Table S3: Regression analysis of the data of individual phenolic compounds adsorbed per mass of β-glucan (mg g^−1^) in gastric and intestinal fluid electrolyte solutions. Comparison of 15 and 30 mg L^−1^ of initial β-glucan concentration. Table S4: Multiple regression analysis of the data of individual phenolic compounds adsorbed per mass of β-glucan (mg g^−1^) in gastric and intestinal fluid electrolyte solutions, with 15 and 30 mg L^−1^ of initial β-glucan concentration, with different volumes of polyphenol extract (100, 200 and 300 μL).

## Sample Availability

Samples of the compounds belonging to flavan-3-ol, phenolic acid, flavonol, dihydrochalcone subgroups are available from the authors.
