# Supplementary material for: Simulated Gastric and Intestinal Fluid Electrolyte Solutions as an Environment for the Adsorption of Apple Polyphenols onto β-Glucan
Source: Molecules. 2022 Oct 8;27(19):6683. doi: 10.3390/molecules27196683 (PMC9570717; doi:10.3390/molecules27196683)
Supplement: Supplementary file 1 [file molecules-27-06683-s001.zip › molecules-1943240-supplementary.pdf]

Table S1 Phenolic compounds from peel and flesh of apples detected before the adsorption, and after the adsorption in the gastric and intestinal fluid electrolyte solutions, with maximums of UV/Vis spectra

			λ_{max}	
**peak assignment**	**phenolic compound**	**phenolic compound subclass**	**before and after adsorption in the gastric fluid electrolyte solution**	**after the adsorption in the intestinal fluid electrolyte solution**
		**Peel**		
1	procyanidin B1	flavan-3-ol	278	
2	(+)-catechin	flavan-3-ol	276	
3	procyanidin B2	flavan-3-ol	278	
4	chlorogenic acid	phenolic acid	sh 292, 326	sh 278, 326
5	chlorogenic adcid isomer 2*	phenolic acid	sh 292, 328	sh 278, 330
6	(-)-epicatechin	flavan-3-ol	278	
8	quercetin-3-galactoside	flavonol	256,356	256,356
9+10	quercetin-3-glucoside + quercetin-3-rutinoside*	flavonols	256,356	256,356
11	quercetin derivative 1*	flavonol	256,356	256,356
12	quercetin derivative 2*	flavonol	254,356	254,356
13	quercetin derivative 3*	flavonol	262,356	268,352
14	quercetin-3-xyloside	flavonol	256,356	256,354
15	quercetin-3-rhamnoside	flavonol	258,352	256,352
	phenolic acid isomer 1*	phenolic acid		316
		**Flesh**		
1	procyanidin B1	flavan-3-ol	278	
2	(+)-catechin	flavan-3-ol	276	
3	procyanidin B2	flavan-3-ol	278	
4	chlorogenic acid	phenolic acid	sh 292, 326 #	sh 278, 326
5	chlorogenic adcid isomer 2*	phenolic acid	sh 292, 328	sh 278, 330
7	phloretin-2-xyloglucoside*	dihydrochalcone	274	272
	phenolic acid isomer 1*	phenolic acid		316

* Tentative identification

#chlorogenic acid detected before the adsorption, but not after the adsorption in the gastric fluid electrolyte solution

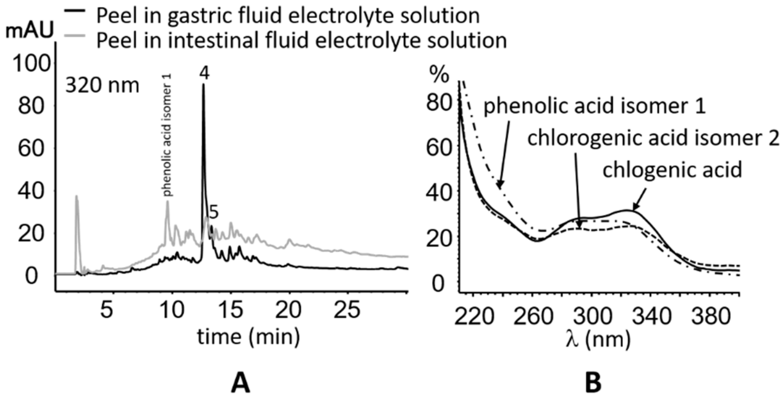

Figure S1. A) the chromatogram of apple peel after the adsorption in the simulated gastric and intestinal fluid electrolyte solutions scanned at 320 nm with detected phenolic acids. B) UV/Vis spectra of phenolic acids. Detected phenolic acids. phenolic acid isomer 1*, 4 – chlorogenic acid, 5 – chlorogenic acid isomer 2*. * - tentative identification

Table S2. Regression analysis of the data of individual phenolic compounds adsorbed per mass of β-glucan (mg g^{-1}). Comparison of gastric and intestinal fluid electrolyte solutions.

Term	Coefficient	Standard error of Coefficient	P-Value
gastric fluid electrolyte solution case	0.03497	0.00929	0.000
intestinal fluid electrolyte solution case	0.1812	0.0114	0.000

In equation $y = ax_1 + bx_2$ the x_1 and x_2 are volumes of extract (μl) in gastric or intestinal fluid electrolyte solution cases, respectively. Coefficients are slopes a and b. The differences of slopes are statistically significant.

Table S3. Regression analysis of the data of individual phenolic compounds adsorbed per mass of β-glucan (mg g^{-1}) in gastric and intestinal fluid electrolyte solutions. Comparison of 15 and 30 mg l^{-1} of initial β-glucan concentration

Term	Coefficient	Standard error of Coefficient	P-Value
15 mg l^{-1} of β-glucan case	0.1244	0.0113	0.000
30 mg l^{-1} of β-glucan case	0.0626	0.0113	0.000

In equation $y = ax_1 + bx_2$ the x_1 and x_2 are volumes of extract (μl) in 15 and 30 mg l^{-1} of β-glucan cases, respectively. Coefficients are slopes a and b. The differences of slopes are statistically significant.

Table S4. Multiple regression analysis of the data of individual phenolic compounds adsorbed per mass of β-glucan (mg g^{-1}) in gastric and intestinal fluid electrolyte solutions, with 15 and 30 mg l^{-1} of initial β-glucan concentration, with different volumes of polyphenol extract (100, 200 and 300 μl)

**Term indicators**	**Coefficient**	**Standard error of Coefficient**	**P-Value**
intestinal fluid electrolyte solution	-0.0288	0.0100	0.004
15 mg l^{-1}	0.02690	0.00497	0.000
intestinal fluid, peel phenolics, 15 mg l^{-1}	0.1328	0.0116	0.000
intestinal fluid q3gluq3rut,15 mg l^{-1}	0.3688	0.0265	0.000
intestinal fluid, chlorogenic acid, 30 mg l^{-1}	0.1289	0.0172	0.000
intestinal fluid, chlorogenic acid,15 l^{-1}	0.5195	0.0239	0.000
intestinal fluid, quercetin-3-rhamnoside,15 mg l^{-1}	0.5735	0.0239	0.000
intestinal fluid, quercetin-3-galactoside,15 mg l^{-1}	0.1650	0.0265	0.000
(-)-epicatechin	0.1406	0.0161	0.000
quercetin-3-galactoside	0.0427	0.0133	0.001
q3glu q3rut	0.0410	0.0133	0.002
quercetin derivative 2	0.0343	0.0119	0.004
quercetin-3-xyloside	0.0359	0.0119	0.003
quercetin-3-rhamnoside, 30 mg l^{-1}	0.1363	0.0163	0.000

Standard deviation of residuals= 11.92, r^2= 90.97

In equation $y= ax_1 + bx_2 +cx_3+\ldots$, the $x_1,x_2\ldots$ are volumes of extract (μl) in the indicated cases. The x_a are zeroed for all other cases. The coefficients are the slopes a, b…. The coefficients are statistically significant.

Note that the slope (coefficient) is negative for the intestinal fluid variable. The explanation is that overall increase is captured by the 15 mg l^{-1} in peel cases and by certain polyphenol cases.
